# Accurate Description of Protein–Protein Recognition and Protein Aggregation with the Implicit-Solvent-Based PACSAB Protein Model

**DOI:** 10.3390/polym13234172

**Published:** 2021-11-29

**Authors:** Agustí Emperador

**Affiliations:** Department of Physics, Universitat Politècnica de Catalunya, B4-B5 Campus Nord, Jordi Girona 1-3, 08034 Barcelona, Spain; agusti.emperador@upc.edu

**Keywords:** protein association, protein aggregation, molecular recognition, implicit solvent, intrinsically disordered proteins, coarse-grained protein models

## Abstract

We used the PACSAB protein model, based on the implicit solvation approach, to simulate protein–protein recognition and study the effect of helical structure on the association of aggregating peptides. After optimization, the PACSAB force field was able to reproduce correctly both the correct binding interface in ubiquitin dimerization and the conformational ensemble of the disordered protein activator for hormone and retinoid receptor (ACTR). The PACSAB model allowed us to predict the native binding of ACTR with its binding partner, reproducing the refolding upon binding mechanism of the disordered protein.

## 1. Introduction

The exponential increase in computational power along with the use of parallelized algorithms for molecular dynamics (MD) simulations has made it possible to reach computational timescales beyond the microsecond, where processes like protein association/dissociation or the full conformational sampling of disordered proteins occur. The achievement of unprecedented timescales has unveiled inaccuracies in the force fields and water models used generally so far, deficiencies that remained unnoticed in shorter timescale simulations.

An important limitation of current explicit solvent models is the inability to predict the correct binding of proteins in molecular dynamics simulations, which leads to non-native binding and aggregation in the simulations. Several works [1,2,3] have shown that current water models and force fields tend to produce binding interfaces between two protein molecules that are known from experiment to be wrong [4] (nonspecific association), overestimating association and thus producing a fraction of monomers much lower than the real, experimentally measured value. This spurious tendency protein association has been related to the general tendency to produce overly collapsed structural ensembles for disordered proteins [5].

Besides these accuracy issues, a simulation of the association of strongly aggregating proteins like the amyloid-β (Aβ) peptide is inaccessible with explicit solvent MD due to the size of the systems that should be used to reproduce the low concentrations (30 μM) at which aggregation begins for these peptides. A system of two Aβ molecules with a concentration of 30 μM involves the use of a simulation box size of 48 nm, which contains around 4 million water molecules. Taking into account that the encounter frequency between two peptides in water is of the order of 10${}^{8}$ s${}^{-1}$ M${}^{-1}$ [6], the average collision time for a concentration of 30 μM is of the order of 10 μs. Simulations of systems involving millions of atoms have been achieved in the submicrosecond timescale [7], but the simulation of such large systems at timescales beyond the microsecond is impossible even with the most powerful supercomputers, leaving as only option the use of the implicit solvent approach [8], which removes from the simulation millions of solvent molecules.

In this work, we apply our PACSAB model [9] to the study of protein aggregation and protein–protein molecular recognition. The PACSAB model is a coarse-grained protein model based on an implicit solvent approach, which uses a highly detailed coarse-grained representation of the amino acid side chains, while keeping an atomistic representation of the backbone in order to describe accurately secondary structure elements. Here, we use this model to study the correlation between helicity and aggregation for the disordered, aggregating peptides Aβ40 and IAPP. After this, we refine the force field of our model to reproduce the monomer–dimer balance of ubiquitin experimentally observed in solutions at 5 mM concentration, to sample the conformational space of the disordered protein activator for hormone and retinoid receptor (ACTR) and to study the mechanism of molecular recognition of ACTR with its binding partner the nuclear coactivator binding domain of the CREB binding protein (NCBD).

## 2. Methods

**Figure 1:**
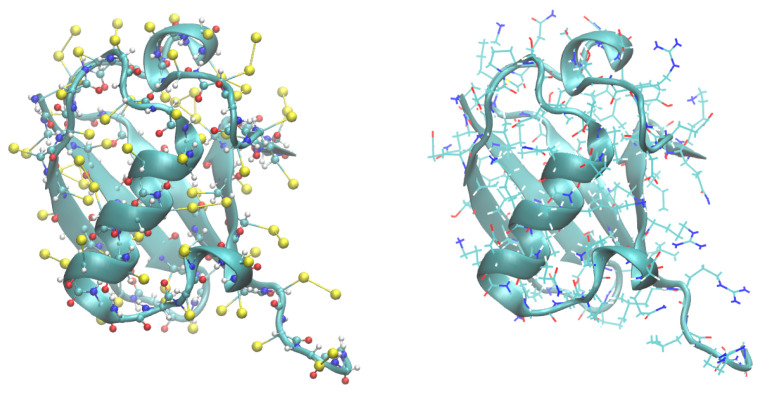
Structure of ubiquitin with the PACSAB model (**left**) compared to the atomistic structure (**right**). A cartoon representation has been used in both cases, plus a bead representation (with the side-chain beads in yellow) in the PACSAB structure.

The details of the implementation of the PACSAB model are explained in our previous works [9,10], where it was used to study protein aggregation and the conformational ensemble of disordered proteins. The pairwise additive coarse-grained side chain and atomistic backbone (PACSAB) protein model uses an atomistic description of the protein backbone, in order to account for hydrogen bonding, which plays a fundamental role in the structural ensemble of disordered proteins, and a coarse-grained representation of the amino acid side chains (see the structure of a ubiquitin molecule with the PACSAB model in Figure 1). To represent the side chains, we used the mapping of the MARTINI model [11], which produces excellent results for proteins embedded in a lipid environment.

The side-chain bead interaction potentials in the PACSAB force field were optimized to produce the correct association/dissociation equilibrium of peptides, while standard coarse-grained (CG) protein models (for a review we refer the reader to [12]) are optimized to stabilize the structure of folded proteins and produce straightforward protein folding. This produces excessively hydrophobic force field parametrizations and a bias of these models towards protein aggregation and structural collapse for disordered proteins. PACSAB was intrinsically designed to prevent this bias [9], while its results are almost as accurate as highly detailed CG models [13,14,15] designed to reproduce the structure of stable proteins [10].

Instead of standard MD, we used the discrete molecular dynamics algorithm (DMD) [16], which allows to use discretized interaction potentials. The DMD algorithm is especially efficient in the case of an implicit solvent description of the system [17,18,19], and has been successfully applied to the study of multiple problems, like flexible protein–protein docking [20] or simulation of conformational transition pathways in proteins [21]. The force field discretization used in DMD does not introduce any bias in the simulations, since the sampling of the simulation and the thermodynamics of the system under consideration do not change when the particle–particle interaction potentials are discretized.

To model a protein solution we used a system of 2 molecules in a simulation box with periodic boundary conditions whose side length *L* is that corresponding to the desired concentration *C*, which in this case is $C=2/L^{3}$. In order to have more statistics of association and dissociation events and reach a stationary regime, we always run several simulations starting from different relative positions of the two molecules.

For the simulations of the stability of a complex, we ran a short simulation with restraints between all the $C_{\alpha }$ of the complex in order to relax the side chains, whose position of minimum potential energy in the PACSAB coarse-grained model might differ from the experimental complex structure, due to the remapping of the structure from atomistic to coarse-grained beads. Afterwards, we ran each simulation of the stability of the complex starting from a different snapshot of the relaxation simulation.

The PACSAB force field depends fundamentally on two parameters, which are the strengths of the van der Waals and the solvation terms [9]:

$$V\left(r_{ij}\right)=\omega_{vdW}V_{vdW}\left(r_{ij}\right)+\omega_{solv}V_{solv}\left(r_{ij}\right)$$

The hydrophobicity of the force field increases with $\omega_{vdW}$ and decreases with $\omega_{solv}$. The optimization of the PACSAB force field was made by running series of simulations of the system starting from different initial configurations, each series with a certain value of the force field parameter to be optimized.

## 3. Results

We used the force field parametrization of the original PACSAB work [9] to simulate the association of two highly hydrophobic, aggregating peptides of high biomedical interest: the 40-residue-long Aβ40 and the 37-residue-long IAPP. Amyloid-β peptides, whose different alloforms are produced from cleavage of the amyloid precursor protein, form amyloid plaques in the brain of people with Alzheimer’s disease, and the oligomers of Aβ are considered to be the main neurotoxic agent in Alzheimer’s disease [22]. Likewise, early oligomerization of IAPP is responsible for β-cell death in the pancreas [23] in Type II diabetes. It was found in experimental studies by Nerelius et al. [24] that stabilization of the α-helical structure reduces the neurotoxicity and the high aggregation propensity of the Aβ40 peptide, which is disordered in aqueous environment but shows an α-helical structure when embedded in a lipid-like hydrophobic environment. To stabilize the α-helix structure, they used ligands known to bind the 13–26 section of the sequence of the Aβ40 of the peptide, fixing its conformation to α-helix.

**Figure 2:**
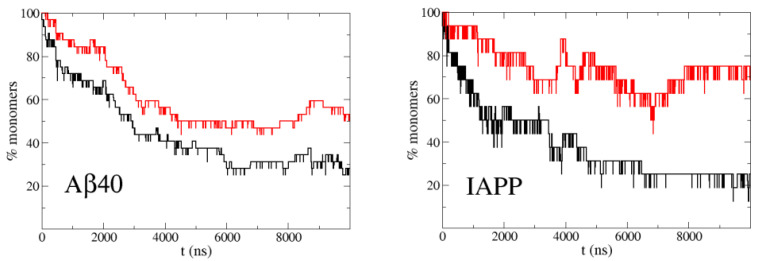
Percentage of monomers in simulations of the highly aggregating peptides Aβ40 at 30 μM concentration (**left**) and IAPP at 100 μM (**right**) with the original PACSAB force field parametrization (black line) and with the new PACSAB force field (red line).

It is known from experiments that Aβ40 at a concentration of 30 μM, roughly one half of the molecules forms oligomers and the other half remains monomeric [25]. For IAPP this happens at a concentration around 100 μM [26]. In order to have a high statistics of association/dissociation events, which are very infrequent due this low concentration (30 μM), we ran 32 simulations of a system of two Aβ40 molecules in a simulation box of 48 nm, corresponding to this concentration, to sample the percentage of monomers. The starting conformation of each simulation was different and the two molecules were extended and far apart. In the case of IAPP at 100 μM, we made 16 simulations with the corresponding box size of 32 nm. Figure 2 shows the evolution of the percentage of monomers along the simulations. A stationary regime is reached in less than 10 μs of trajectory, due to the fast diffusion of the peptides in the simulation with PACSAB and the subsequent increase in the frequency of collisions between molecules. This is possible thanks to the lack of friction with solvent molecules, allowing fast diffusion and a much faster sampling than in explicit solvent simulations, with a speedup factor of several orders of magnitude for implicit solvent simulations of systems without kinetic barriers [27].

Motivated by the experimental observations of Nerelius et al. [24], we wanted to study the effect of helicity on the aggregation dynamics. For this reason we ran simulations with the new PACSAB parametrization [10], which allowed a more realistic description of disordered proteins by including a more hydrophobic interaction between residues close in sequence, which favored the formation of local hydrogen bonds and α-helix structure. With this parametrization the helicity became 50% for Aβ40, while with the original parametrization it was 10%. Figure 2 shows a strong reduction in the aggregation propensity of both Aβ40 and IAPP compared to the simulations with the original PACSAB force field.

**Figure 3:**
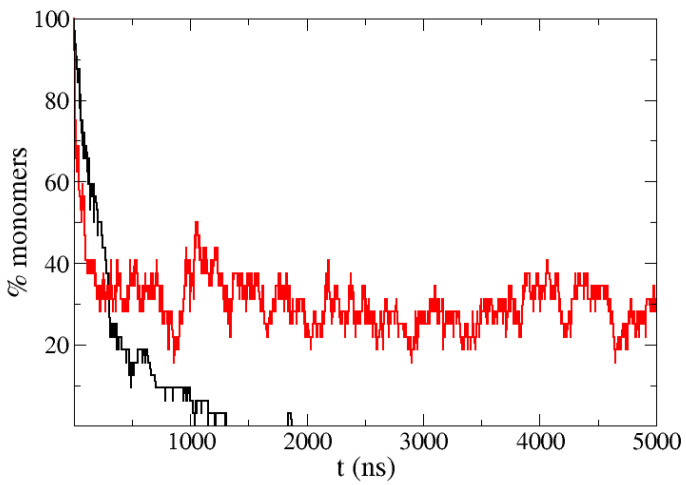
Percentage of monomers in simulations of the highly aggregating peptide Aβ40 at very high concentration (0.3 mM) with the normal PACSAB force field parametrization (black line) and when α-helix structure is restrained in the 13-26 section of the sequence of Aβ40 (red line).

To get stronger evidence of the connection between helicity and decrease in aggregation, we addressed the extreme case of a solution of Aβ40 at 0.3 mM, a concentration 10 times higher. Figure 3 shows that the Aβ40 molecules rapidly aggregate and the monomers disappear in the simulations. We made another set of simulations restraining the α-helix structure in the 13–26 section of the sequence of Aβ40 to mimic the action of the ligands used in the experimental studies [24], and we found that even at such high concentration, around 30% of the molecules remain momomeric in the stationary regime.

**Figure 4:**
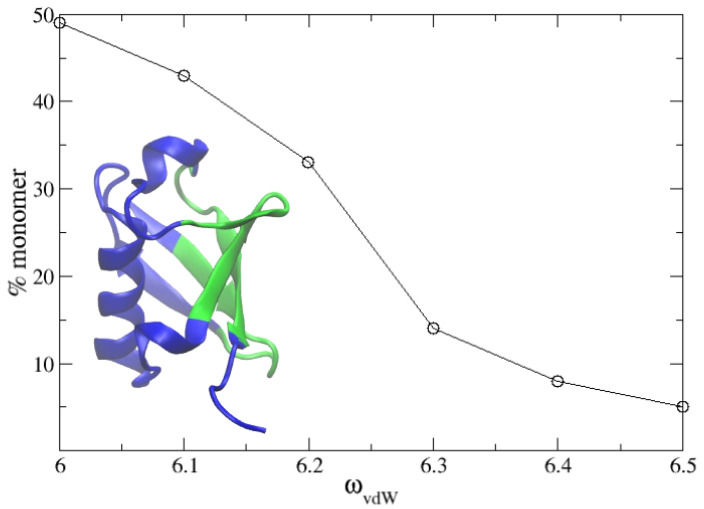
Percentage of solute remaining in monomeric state as a function of the van der Waals term of the PACSAB parametrization in the simulations of a 5 mM solution of ubiquitin. The structure of the protein is shown, highlighting in green the binding interface known from experiment.

In a recent work, we studied the dimerization and molecular recognition of ubiquitin using explicit solvent molecular dynamics simulations [3]. Here, we wanted to test our PACSAB model in this system at the concentration of 5 mM used in the experiments [4]. We refined the parametrization of the new PACSAB force field [10] in order to reproduce the 50% percentage of monomer in a solution of ubiquitin at a 5 mM concentration. We show in Figure 4 the dependence of the percentage of monomers on the force field parameter $\omega_{vdW}$. The refinement involved eight 2 μs long simulations for each value of $\omega_{vdW}$. In the figure, we show the experimentally known [4] binding interface of ubiquitin. This optimization of the force field just produced a slight increase of 5% in $\omega_{vdW}$ with respect to its initial value.

**Figure 5:**
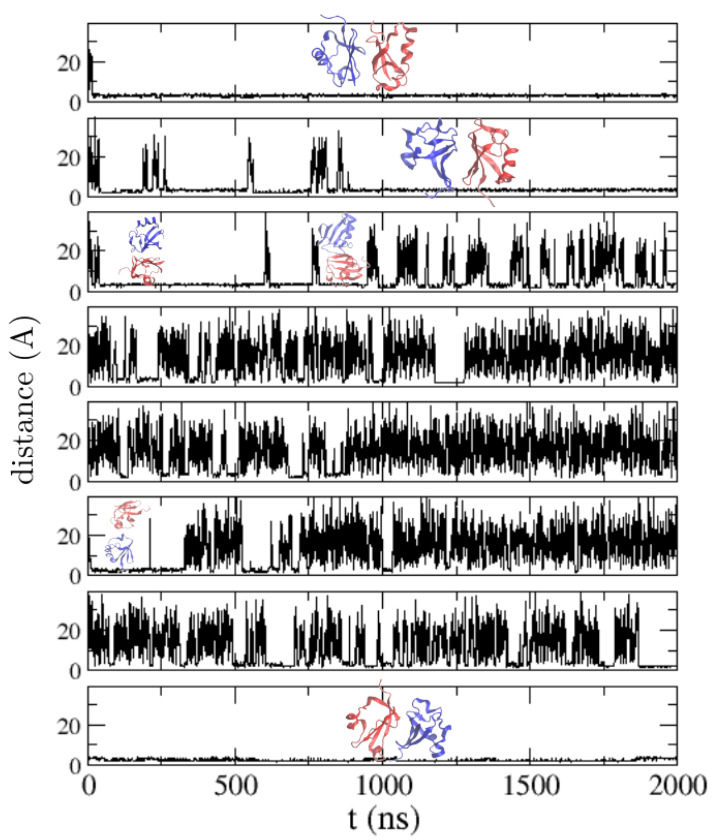
Minimum distance between two ubiquitin molecules in molecular dynamics simulations of a solution of ubiquitin at a concentration of 5 mM with the PACSAB model in the 8 trajectories produced. The most stable bound configurations found during the simulations are shown.

**Figure 6:**
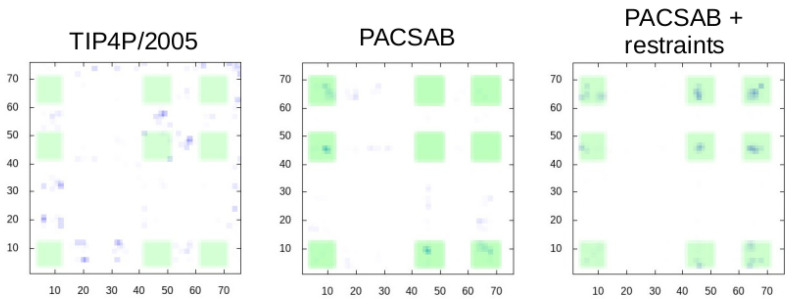
Intermolecular contact maps for the trajectories obtained with explicit solvent MD simulation with the TIP4P/2005 water model (**left**), with the optimized PACSAB force field parametrization (**center**) and with the optimized PACSAB force field parametrization and intramolecular restraints (**right**). X, Y axes are the residue number along protein sequence. The regions where contacts are found in the NMR experiments are highlighted in green.

We show in Figure 5 the eight trajectories obtained with this force field parametrization, showing the most stable bound configurations, and in Figure 6 the intermolecular contact map sampled along these trajectories. It is known from NMR-based experimental studies by Liu et al. [4] that ubiquitin forms transient low-affinity noncovalent dimers defined by a large interface where many relative orientations are possible [1]. The binding interface is the beta-sheet surface of ubiquitin, formed by the residues 4–12, 42–51 and 62–71. We have highlighted in the contact maps (Figure 6) the regions defined by these sequence regions. In our previous work we found that TIP4P/2005 [28] was the water model that produced the best results in explicit solvent MD simulations. TIP4P/2005 reproduced correctly the high diversity of relative positions of the two bound ubiquitin molecules, but failed to predict the native binding interface, as observed in Figure 6. An analysis of the contact maps reveals that the PACSAB model better predicts the native binding interface than explicit solvent simulations with TIP4P/2005, although many contacts with residues outside the experimental binding interface are still formed.

We found in our simulations that this parametrization fails to keep the experimental structure of the ubiquitin molecules, deforming them along the simulation. We wanted to assess if this deformation increases non-native binding outside the experimental interface. With this purpose, we ran eight simulations with the same force field parametrization and starting configurations, but adding an intramolecular-structure-based potential that fixes the $C_{\alpha }$–$C_{\alpha }$ distances inside each molecule, in order to stabilize the ubiquitin structure in its experimental native conformation. The contact map obtained in this case, shown in the right hand side of Figure 6, shows all the contacts inside the region compatible with the experimental interface, meaning that no contacts are made with residues outside this interface.

That point we had found that our parametrization of PACSAB is able to reproduce accurately the dimerization of a stable protein, but we wanted to test it for a protein of a completely different type. We chose as system of study the 46-residue-long intrinsically disordered protein activator for hormone and retinoid receptor (ACTR), which has a very low propensity to aggregation due to its highly hydrophilic sequence. Best et al. [5] studied the conformational ensemble of this disordered protein, finding that best results were obtained with the TIP4P/2005 water model, although the ensemble was still too collapsed when comparing to the radius of gyration of 2.5 nm measured experimentally by SAXS [29]

**Figure 7:**
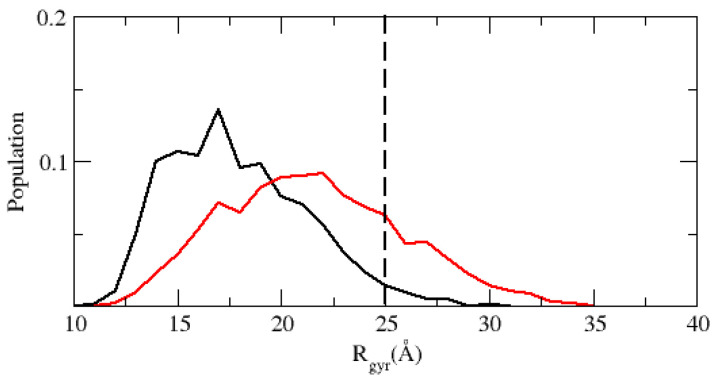
Distribution of radii of gyration in the simulations of the disordered protein ACTR in explicit solvent MD simulations with TIP4P/2005 water (black line) and with the PACSAB model (red line), using the same parametrization used in the simulations of ubiquitin dimerization. The vertical dashed line marks the experimental estimation of the radius of gyration of ACTR.

We simulated the disordered protein ACTR with different values of $\omega_{vdW}$ as we did for the ubiquitin solution, finding that as the hydrophobicity is reduced (decreasing $\omega_{vdW}$) the conformational ensemble is less collapsed. We found that the PACSAB parametrization that gives better results for the ubiquitin solution better approaches the experimental radius of gyration than explicit solvent simulations with TIP4P/2005 water (see Figure 7), although the radius of gyration distribution obtained for this parametrization of the force field is still below the value estimated from experiments.

Finally we wanted to assess the ability of PACSAB to describe protein–protein recognition in the case of a disordered protein. We took as case of study the binding of ACTR to its binding partner nuclear coactivator binding domain of the CREB binding protein (NCBD), a process that involves the refolding of ACTR into the helical structure it shows in the complex ACTR:NCBD, and the recognition of the binding interface. The process of refolding of a disordered protein into its bound stable structure upon binding follows a mechanism that is generally a mixture of the two ideal cases of induced fit and conformational selection [30]. In the induced fit mechanism, the protein refolds in contact with the receptor until it adopts the correct conformation and binds to the receptor, while in the conformational selection mechanism the disordered protein can sample multiple conformations and is able to bind to the receptor when it adopts the correct conformation.

**Figure 8:**
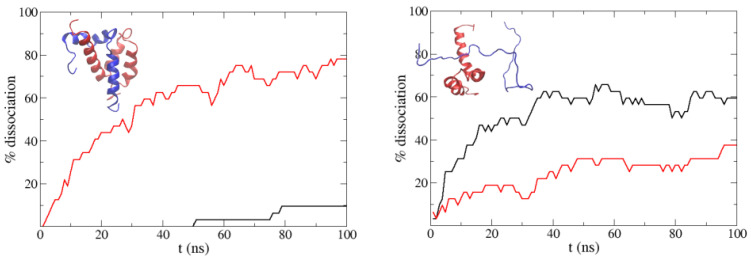
Percentage of dissociated molecules along the simulations starting from the experimental ACTR:NCBD complex (**left**) and from a non-native bound structure (**right**) using the normal PACSAB force field (black line) and when hydrogen bonding is removed (red line). The experimental ACTR:NCBD complex is shown in the left panel (ACTR is the blue molecule).

Before trying to reproduce the binding of the two proteins, we started analyzing the effect of the α-helix secondary structure of ACTR on the stability of the ACTR:NCBD complex (shown in the left panel of Figure 8; ACTR is the blue molecule). With this purpose, we ran a set of 32 simulations starting from relaxed snapshots of the experimental complex (see Section 2 above), and an extra set “unplugging” hydrogen bonding in our force field. In order to reduce the configuration space of our system and focus on the behavior of ACTR, in all our simulations the NCBD molecule was fixed in its experimental binding conformation by fixing the distances between the $C_{\alpha }$ of the NCBD. In the case of this complex, we had to increase the hydrophobicity of the force field because we found immediate dissociation when using the normal PACSAB parametrization. So we increased $\omega_{vdW}$ until the complex did not dissociate before 100 ns.

Figure 8 shows the percentage of dissociation along the simulations: we observe that when hydrogen bonding is removed, ACTR dissociates faster from NCBD. We also ran simulations starting from non-native bound structures of NCBD with unfolded ACTR (these configurations were obtained from a long simulation with periodic boundary conditions). In this case, we found an interesting trend: if hydrogen bonding is included, dissociation is faster than if it is not included. Having in mind that protein aggregation results from accumulated non-native binding, this trend is consistent with the decrease of aggregation we found for Aβ40 and IAPP, when we used a force field parametrization which favored the α-helix secondary structure of the peptides.

**Figure 9:**
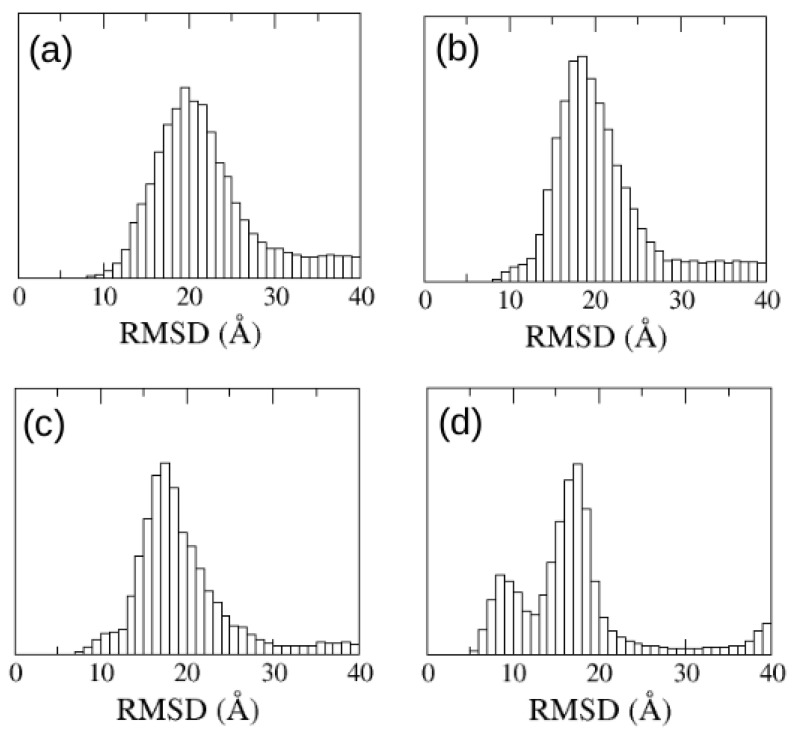
Distribution of RMSD with respect to the experimental ACTR:NCBD complex of the configurational ensemble sampled along the MD trajectories for the simulations starting from (**a**) unfolded ACTR, (**b**) ACTR with native helicity, (**c**) unfolded ACTR with enhanced hydrogen bonding, and (**d**) ACTR with native helicity and enhanced hydrogen bonding.

Next we ran 16 simulations of 2 μs starting from the ACTR and NCBD molecules far apart, as we had done for the aggregating peptides. We ran one series starting from the unfolded, extended ACTR, and another series with ACTR in the folded conformation it shows in the ACTR:NCBD experimental complex. In that case, we observed that the helicity of the ACTR molecule was rapidly lost and became a random coil due to the interaction with the solvent, as should be expected from an intrinsically disordered protein. We show in Figure 9 the distribution of RMSD with respect to the experimental ACTR:NCBD complex for different sets of simulations. In the case of simulations starting from the unfolded ACTR molecule no peak is found at RMSD < 10 Å, meaning that native binding is scarcely found. In the case of the simulations starting from ACTR in an helical conformation, we observed a small bump around 10 Å, which corresponds to some configurations where ACTR contacts NCBD near the correct binding interface, but ACTR is highly unfolded due to the loss of helicity along the simulation.

Our results indicate that the α-helix structure favors the stability of bound configurations near the native binding interface. Previous experimental studies on the binding of ACTR with NCBD [31] indicated that a preformed α-helix structure in ACTR enables molecular recognition with NCBD. This motivated us to make a new set of 2 μs long simulations with a slightly more hydrophobic parametrization of the force field and an increased hydrogen bond energy, in order to boost the helicity of the ACTR molecule. We ran, again, one set 16 simulations starting from unfolded conformations of ACTR and another set of 16 simulations starting from the helical folded structure. We found that when starting from folded ACTR, 30% of the sequence remained in α-helix, while with the former parametrization it was only 20%.

**Figure 10:**
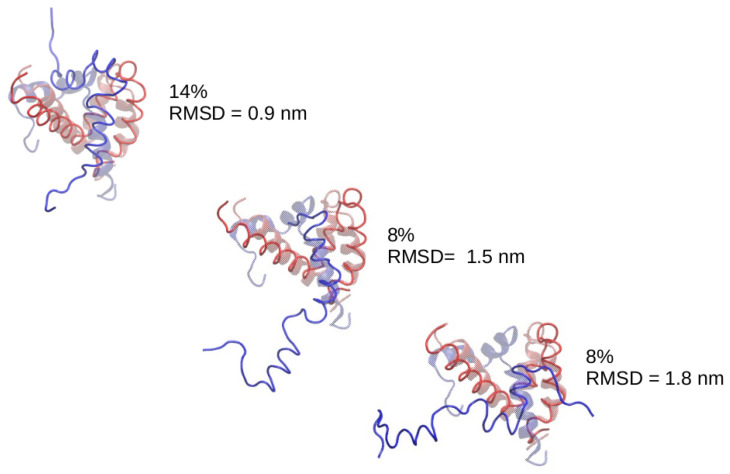
Central structure of the most populated clusters in the set of simulations with enhanced hydrogen bonding. The ACTR molecule is shown in blue and the structures have been superimposed to the experimental complex (transparent).

In the case of the simulations starting from ACTR molecules completely unfolded we just found a bump near RMSD = 10 Å, but for the simulations starting from ACTR in helical configuration, we found a clear peak around RMSD = 9 Å. We made a clustering analysis of the conformations sampled by the system along this set of simulations, finding that the most populated cluster is centered around a bound structure within 9 ÅRMSD to the experimental ACTR:NCBD complex. We show in Figure 10 the most populated clusters, indicating which percentage of the sampled configurations belongs to each cluster. For better interpretation, the central structure of each cluster has been superimposed to the experimental complex. An examination of the central structure of the main cluster reveals that, despite having an RMSD of 9 Åwith respect to the experimental complex, it shows the correct location of ACTR on the NCBD molecule, therefore this corresponds indeed to native binding. The high value of the RMSD for this native cluster is due to the high flexibility of ACTR (the RMSD of ACTR with respect to its own bound crystallographic structure is 11 Å). Consequently, we found that the PACSAB force field slightly modified to enhance α-helix structure is able to predict the native conformation of the ACTR:NCBD complex.

## 4. Conclusions

We found in this work that PACSAB, a coarse-grained protein model based on the implicit solvent approach, is able to predict the correct binding interface in the binding of both a stable protein (ubiquitin) and a disordered protein (ACTR), improving the results of explicit solvent atomistic simulations for these systems. We exploited in our simulations the extremely fast sampling obtained with the implicit solvent approach in protein solutions to study computationally long timescale phenomena inaccessible with standard explicit solvent molecular dynamics. We could adjust the PACSAB force field in order to predict both the binding interface of ubiquitin and the conformational space of the disordered protein ACTR.

We used the PACSAB model to study how the oligomerization of aggregating peptides depends on its abundance of α-helix structures, finding results in complete agreement with the experimental evidence that fixing the helical conformation of Aβ40 strongly decreases its oligomerization. In the case of the disordered protein ACTR, we found that the PACSAB model, apart from removing the undue structural collapse found in explicit solvent simulations, is able to predict both the structure of ACTR in the ACTR:NCBD complex and the native binding interface with its binding partner NCBD, reproducing the refolding and molecular recognition mechanism of this disordered protein.

Altogether, our results indicate that in the case of disordered proteins, the α-helix reduces non-native binding and increases native binding, in agreement with previous experimental evidence. The accuracy of the PACSAB model in describing these phenomena suggests that accurate coarse-grained protein models based on the implicit solvent approach can be successful in predicting molecular recognition of both stable and disordered proteins, encouraging its future application to more study cases. 

## Data Availability

Data is contained within the article.
